# Interpretable clinical phenotypes among patients hospitalized with COVID-19 using cluster analysis

**DOI:** 10.3389/fdgth.2023.1142822

**Published:** 2023-04-11

**Authors:** Eric Yamga, Louis Mullie, Madeleine Durand, Alexandre Cadrin-Chenevert, An Tang, Emmanuel Montagnon, Carl Chartrand-Lefebvre, Michaël Chassé

**Affiliations:** ^1^ Department of Medicine, Centre Hospitalier de l’Université de Montréal (CHUM), Montréal, QC, Canada; ^2^ Centre de Recherche du Centre Hospitalier de l'Université de Montréal (CRCHUM), Montréal, QC, Canada; ^3^ Department of Medical Imaging, CISSS Lanaudière, Université Laval, Joliette, QC, Canada; ^4^ Department of Radiology and Nuclear Medicine, Centre Hospitalier de l’Université de Montréal (CHUM), Montréal, QC, Canada

**Keywords:** unsupervised learning (UL), COVID-19, clinical phenotypes, machine learning interpretability, data visualization

## Abstract

**Background:**

Multiple clinical phenotypes have been proposed for coronavirus disease (COVID-19), but few have used multimodal data. Using clinical and imaging data, we aimed to identify distinct clinical phenotypes in patients admitted with COVID-19 and to assess their clinical outcomes. Our secondary objective was to demonstrate the clinical applicability of this method by developing an interpretable model for phenotype assignment.

**Methods:**

We analyzed data from 547 patients hospitalized with COVID-19 at a Canadian academic hospital. We processed the data by applying a factor analysis of mixed data (FAMD) and compared four clustering algorithms: k-means, partitioning around medoids (PAM), and divisive and agglomerative hierarchical clustering. We used imaging data and 34 clinical variables collected within the first 24 h of admission to train our algorithm. We conducted a survival analysis to compare the clinical outcomes across phenotypes. With the data split into training and validation sets (75/25 ratio), we developed a decision-tree-based model to facilitate the interpretation and assignment of the observed phenotypes.

**Results:**

Agglomerative hierarchical clustering was the most robust algorithm. We identified three clinical phenotypes: 79 patients (14%) in Cluster 1, 275 patients (50%) in Cluster 2, and 203 (37%) in Cluster 3. Cluster 2 and Cluster 3 were both characterized by a low-risk respiratory and inflammatory profile but differed in terms of demographics. Compared with Cluster 3, Cluster 2 comprised older patients with more comorbidities. Cluster 1 represented the group with the most severe clinical presentation, as inferred by the highest rate of hypoxemia and the highest radiological burden. Intensive care unit (ICU) admission and mechanical ventilation risks were the highest in Cluster 1. Using only two to four decision rules, the classification and regression tree (CART) phenotype assignment model achieved an AUC of 84% (81.5–86.5%, 95 CI) on the validation set.

**Conclusions:**

We conducted a multidimensional phenotypic analysis of adult inpatients with COVID-19 and identified three distinct phenotypes associated with different clinical outcomes. We also demonstrated the clinical usability of this approach, as phenotypes can be accurately assigned using a simple decision tree. Further research is still needed to properly incorporate these phenotypes in the management of patients with COVID-19.

## Introduction

Patients affected by coronavirus disease 2019 (COVID-19) have shown significant clinical heterogeneity and variability in disease trajectory (1). Clinical phenotypes are homogeneous disease subgroups with distinct clinical features (2). Beyond descriptive categories, phenotypes hold prognostic value. Well-established phenotypes are needed at the bedside for proper patient classification, clinical trial enrolment, disease prognostication, and treatment personalization.

Since the first case description of COVID-19, various phenotypes have emerged, each of which uses various layers of clinical information. Two phenotypes have been described based on lung mechanics and radiological findings (3, 4). Others have focused on disease complications—as such, a hypercoagulable phenotype has been observed, prompting recommendations for intensified antithrombotic therapy (5–7). Most of these phenotypes failed to fully describe the complexity of the disease, as they focused on characterizing only one dimension of the clinical presentation. Being mainly derived from clinical observation, the reliability and the methodology of these first phenotyping efforts have been put into question (8, 9).

Consequentially, interest arose in applying data-driven methodologies to phenotyping. Clustering is an unsupervised machine-learning (ML) method used to identify homogeneous groups within a heterogeneous dataset. These methods are hypothesis-agnostic and rely solely on the assumption that clinical patterns lie within the data (10). This method has been previously used for disease phenotyping, such as chronic obstructive pulmonary disease (COPD) (11) and sepsis (12). Similarly, several authors have recently used clustering to identify clinical phenotypes in patients with COVID-19 (13–21).

Methodologically, these efforts did not include unprocessed imaging data, which are a key determinant of COVID-19 prognostication (22). Additionally, none have focused on interpretability, hampering the implementation of data-driven phenotypes at the bedside and their complete understanding by clinicians.

Here, we aimed to identify COVID-19 phenotypes at patient presentation using multimodal real-world clinical and medical imaging data, and assess their association with three clinical outcomes: mechanical ventilation (MV), intensive care unit (ICU) admission, and hospital mortality. We hypothesized that imaging data were crucial in enhancing the reliability of clustering efforts in the context of COVID-19, and aimed to facilitate the interpretation and assignment of patients to one of the identified phenotypes through data visualization and decision tree modelling, respectively (23).

## Methods

### Data sources

We used real-world data extracted from a clinical data system comprising relevant information from all COVID-19-related hospitalizations at the Centre for the Integration and Analysis of Medical Data (CITADEL) of the Centre Hospitalier de l'Université de Montréal (CHUM), a Canadian academic quaternary center. The analytical dataset contained de-identified data for over 1,100 patients hospitalized with COVID-19, including demographics, comorbidities, laboratory results, vital signs, drugs, medical procedures, frontal chest radiographs (CXR), and clinical outcomes. The raw data were managed using SQLite 3, and further data processing was conducted using Python version 3.7 and R version 4.0.3. Additional details regarding the initial data processing are provided (see S1 text, Supplementary Methods).

### Study population

We included all unique adult hospitalizations (≥18 years of age) for COVID-19 from January 1, 2020, to January 30, 2021, for which a chest x-ray was available within 24 h of admission. A COVID-19 hospitalization episode was defined as hospitalization within seven days of a positive SARS-CoV-2 PCR result. The Institutional Review Board of the CHUM (Centre Hospitalier de l'Université de Montréal) approved the study, and informed consent was waived because of its low risk and retrospective nature.

### Imaging data processing

Imaging data were obtained in DICOM format, and frontal CXR (posteroanterior and anteroposterior) were processed, discarding lateral CXR. Lung opacities observed on CXR were manually annotated with bounding boxes by a board-certified radiologist using a bounding box annotation software (24). This annotation method is recognized by the Radiological Society of North America (RSNA) and is the annotation methodology of choice for all deep-learning challenges involving image detection (25, 26). Bounding boxes are rectangular or squared delimitations of the opacities found on a chest radiograph reported with a scaled width and length. Hence, from the manual annotation, we derived the number of opacities and the total size of opacities as a relative percentage of the total surface of the image.

### Variables selection and feature engineering

A total of 160 candidate variables were extracted from the analytical dataset. We provided the list of those variables in the supplementary material (see Supplementary Table S1). For each variable, we exclusively used the first recorded value within the first 24 h of admission. We then excluded 56 variables for which more than 25% of observations were missing. The remaining missing variables were imputed using all available features, excluding the clinical outcomes (ICU admission, mechanical ventilation, and death). We used classification and regression tree (CART) single mean imputation, a robust method against outliers, multicollinearity, and skewed distributions, which is simple to implement in a real-world setting (27). We selected this imputation method because of its lower computational cost, which makes it more feasible to implement compared to methods with superior performance, such as Expectation Maximization (EM) and Multiple Imputation (MI) (28). We computed the Medicines Comorbidity Index (MCI), a metric to assess multimorbidity that has shown epidemiological value similar to the Charlson Comorbidity Index (CCI) (29). We relied on MCI instead of CCI because comorbidities were not systematically recorded in our database, but medications were (see Supplementary Table S1). MCI was computed at the time of study enrollment using only the data available upon clinical presentation. Table 1 summarizes the final set of variables included in the analysis with their respective subdomains of interest.

**Table 1:** Final set of variables and respective subdomains.

Subdomain	Clinical variable
Demographic	Age, sex, comorbidities (MCI)
Hemodynamic	SBP, DBP, HR, Anion Gap
Respiratory	FiO2, SpO_2_
Imaging	Opacities number, Opacities size
Hematologic	Neutrophils, Lymphocytes
Inflammatory/Thrombotic	Mean Platelet Volume (MPV), Temperature
Renal	Creatinine, Sodium, Potassium, Bicarbonate

### Cluster analysis

Before applying the clustering algorithms, we processed our dataset, log-transformed skewed continuous variables (skewness > 0.5), and excluded highly correlated variables (correlation > 0.8). We then obtained the principal components *via* factor analysis of mixed data (FAMD) (30) for all our observations. The only excluded variable based on high correlation was the total white blood cell count (WBC), which was correlated with the absolute neutrophil count (ANC).

We compared four clustering algorithms: k-means, partitioning around medoids (PAM), and divisive and agglomerative hierarchical clustering. We used three internal validation metrics (connectivity, Dunn index, and average silhouette width) and four stability measures: the average proportion of non-overlap (APN), average distance (AD), average distance between means (ADM), and figure of merit (FOM) to compare the algorithms. The optimal algorithm and number of clusters were then determined by rank aggregation of the ranked lists of each validation metric. We used the optCluster package, an R package facilitating the execution of the aforementioned analyses (31). We have provided additional details regarding our cluster analysis in the Supplementary Material (see the Supplementary S1 Text).

**Figure 1:**
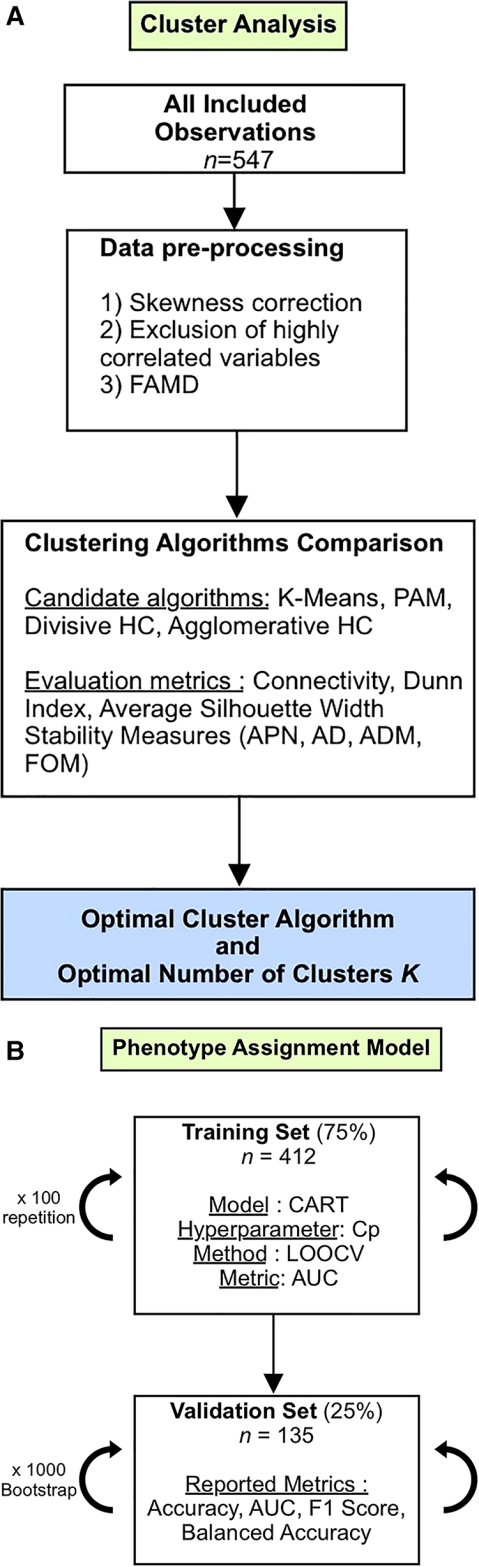
(**A**) Study flowchart (Cluster Analysis). FAMD, factor analysis of mixed data; PAM, partition around medoids; HC, hierarchical clustering; APN, average proportion of non-overlap; AD, average distance; ADM, average distance between means; FOM, figure of merit. (**B**) Study flowchart (Phenotype Assignment Model). CART, classification and regression tree; Cp, complexity parameter; LOOCV, leave-one-out cross-validation; AUC, area under the curve.

A summary of the development process of our clustering algorithm is shown in Figure 1A.

### Clinical outcomes evaluation

We conducted survival analysis using the Kaplan-Meier method to compare clinical outcomes according to clusters. We assessed three clinical outcomes:7-day ICU admission, 7-day mechanical ventilation, and 30-day mortality. To reduce confounding bias, we specifically restricted clinical outcome analysis to patients eligible for MV and ICU admission according to the Physician Orders for Life-Sustaining Treatment (POLST) form. All statistical analyses were performed using R software (version 4.0.3, R Foundation for Statistical Computing), and *p*-value (2-sided) below 0.05 was considered statistically significant.

### Phenotype assignment model and clusters interpretability

To facilitate the interpretation and clinical usability of the obtained clusters, we trained a simple decision tree using CART with the clusters as the predicted outcomes (32). Because the primary objective was to favor interpretability, we did not compare CART with other supervised machine learning models.

We built the CART model using R package caret. All patients included in the study were randomly split into a training set (75%) and a validation set (25%). The model Complexity Parameter (Cp) was tuned using leave-one-out cross-validation (LOOCV) 100 times on the training set, with the area under the curve (AUC) as the evaluation metric. Because this classification problem focuses more on predicted scores than predicted classes, we favored AUC over alternative evaluation metrics.

We validated the model's performance by calculating four macro-averaged classification metrics on 1,000 bootstrap samples from the validation set. We opted for macro-averaging because each class is of equal importance. The computed metrics were AUC, balanced accuracy, accuracy, precision, recall, and F1 score. Subgroup analyses based on the COVID-19 waves during admission were performed. The different steps followed in the modeling process are summarized in Figure 1B.

We determined the most critical variables to discriminate between clusters by conducting a variable importance analysis (VIA) (33). Details regarding the VIA are provided in the Supplementary Material (see Supplementary S1 Text).

Finally, we computed three variables that have recently been associated with COVID-19 mortality: neutrophil-to-lymphocyte ratio (NLR) (34), ratio of peripheral arterial oxygen saturation to the inspired fraction of oxygen (SpO_2_/FiO_2_) (35), and shock index (heart rate/systolic blood pressure) (36). These variables were not used for the clustering effort and were computed strictly for descriptive and interpretability purposes.

### Phenotypes robustness: sensitivity analyses

We conducted a sensitivity analysis to assess whether the removal of imaging data altered the performance of the clustering algorithm. We first compared the clustering results given these two scenarios using the average silhouette width and the adjusted Rand index (37), a measure of the similarity between two data clustering. We then assessed the number of patients who underwent phenotypic reclassification before and after the removal of the imaging data in the clustering algorithm. In other words, we analyzed the characteristics of patients for whom the assigned cluster differed after the removal of imaging data.

## Results

### Study population

**Figure 2:**
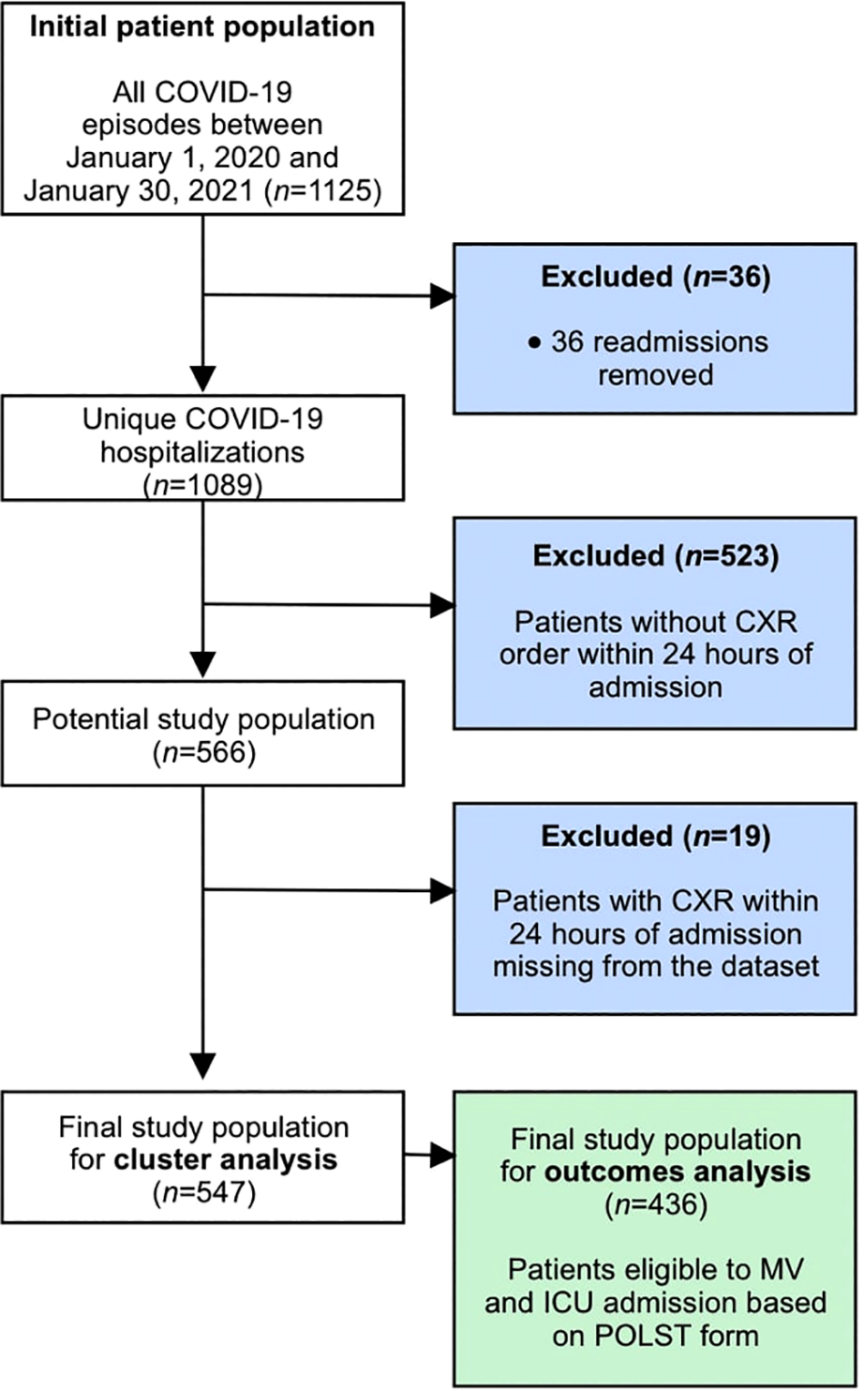
Study inclusion and exclusion criteria.

In total, 1,125 unique COVID-19 hospitalizations were screened. A total of 559 patients were excluded after removing readmissions (*n *= 36), patients without a CXR within 24 h of admission (*n* = 523), and patients for whom clinical data were missing (*n = *19), leaving 547 patients for the cluster analysis (see Figure 2). We have provided details regarding the characteristics of the study cohort (see Table 2). Our population was similar to other cohorts of patients hospitalized with COVID-19 in North America during the first two waves, with a mean age of 69 years, a relatively similar proportion of men and women, and an in-hospital mortality rate of 20% (38).

**Table 2:** Baseline characteristics of the study population.

Characteristics	*n* = 547
Age (years), mean (SD)	66.56 (17.94)
Sex (male), *n* (%)	313 (57.2)
Medicines Comorbidity Index, mean (SD)	2.77 (2.04)
Laboratory results	
Hemoglobin, mean (SD)	127.23 (20.69)
Platelet, median [IQR]	207.00 [157.00, 271.50]
WBC, median [IQR]	6.80 [5.30, 9.85]
Neutrophil count, median [IQR]	5.01 [3.60, 7.60]
Lymphocyte count, median [IQR]	0.95 [0.62, 1.35]
Monocytes count, mean (SD)	0.63 (0.39)
Basophils count, mean (SD)	0.01 (0.03)
Eosinophils count, mean (SD)	0.05 (0.12)
Mean Platelet Volume (MPV), mean (SD)	9.89 (1.33)
Mean Corpuscular Volume (MCV), median [IQR]	89.90 [85.90, 93.85]
Sodium, mean (SD)	137.59 (5.05)
Potassium, mean (SD)	4.00 (0.53)
Bicarbonate, mean (SD)	24.95 (3.76)
Anion gap, mean (SD)	11.05 (3.69)
Creatinine (µmol/L), median [IQR]	78.00 [63.00, 105.00]
Vital Signs	
FiO_2_, median [IQR]	21.00 [21.00, 28.00]
SpO_2_, median [IQR]	95.00 [94.00, 97.00]
Temperature, mean (SD)	36.98 (0.51)
Systolic Blood Pressure, median [IQR]	130.00 [116.00, 144.50]
Diastolic Blood Pressure, median [IQR]	75.00 [68.00, 82.00]
Heart Rate, mean (SD)	92.2 (20.2)
Respiratory Rate, median [IQR]	20.00 [20.00, 24.00]
Medication	
Anticholesterolemic agents, *n* (%)	183 (33.5)
Antihypertensive agents, *n* (%)	241 (44.1)
Bronchodilator agents, *n* (%)	178 (32.5)
Diuretics, *n* (%)	137 (25.0)
Factor Xa Inhibitors, *n* (%)	50 (9.1)
Hypoglycemic agents, *n* (%)	209 (38.2)
Platelet aggregation inhibitors, *n* (%)	150 (27.4)
Imaging Data	
Opacities Numbers	
0, *n* (%)	141 (25.8)
1, *n* (%)	89 (16.3)
2, *n* (%)	279 (51.0)
3, *n* (%)	37 (6.8)
4, *n* (%)	1 (0.2)
Opacities Size (surface area %), mean (SD)	9 (8)
Clinical outcomes	
Length of Stay (days), median [IQR]	8.29 [3.48, 18.20]
Mechanical Ventilation, *n* (%)	48 (8.8)
Wave (1st), *n* (%)	295 (53.9)
ICU admission, *n* (%)	132 (24.1)
Death, *n* (%)	113 (20.7)

### Clinical characteristics of phenotypes

Agglomerative hierarchical clustering was deemed to be the most robust clustering algorithm for our dataset. The optimal number of K clusters was K = 3 (see Supplementary Figure S1), yielding the highest clustering performance, as exhibited by the rank aggregation of the seven internal validation measures (see Supplementary Figure S2, Table S2).

The characteristics of these clusters are summarized in Table 3. As determined through the VIA, the most critical variables for discriminating clusters were MCI, age, opacities size, and absolute neutrophil count (see Supplementary Figure S3).

**Table 3:** Clinical characteristics stratified by clusters.

	Cluster 1	Cluster 2	Cluster 3
*n* (%)	79 (14)	265 (48)	203 (37)
Age (years), mean (SD)	58.81 (15.42)	75.14 (13.31)	57.99 (19.14)
Sex (male), *n* (%)			
Medicines Comorbidity Index (MCI), mean (SD)	2.38 (2.06)	3.93 (1.72)	1.43 (1.50)
Laboratory results			
Hemoglobin, mean (SD)	121.73 (22.82)	124.87 (20.70)	132.48 (19.76)
Platelet, mean (SD)	292.14 (104.41)	213.00 (89.05)	210.50 (87.48)
WBC,^†^ median [IQR]	9.50 [7.35, 14.25]	6.80 [5.30, 9.50]	6.20 [4.90, 8.70]
Neutrophil count, median [IQR]	7.80 [5.64, 12.70]	5.10 [3.63, 7.20]	4.40 [3.18, 6.49]
Lymphocyte count, median [IQR]	0.80 [0.47, 1.10]	0.90 [0.60, 1.30]	1.09 [0.70, 1.59]
MCV, median [IQR]	87.00 [83.05, 90.90]	90.90 [87.35, 94.50]	89.00 [84.70, 93.10]
MPV, mean (SD)	9.43 (1.08)	9.91 (1.37)	10.01 (1.32)
NLR,* median [IQR]	9.91 [5.64, 19.55]	5.62 [3.38, 9.83]	4.33 [2.53, 7.15]
Sodium, mean (SD)	136.91 (5.07)	138.21 (4.74)	137.61 (5.21)
Potassium, mean (SD)	4.05 (0.70)	4.05 (0.51)	3.95 (0.45)
Bicarbonate, mean (SD)	23.80 (4.17)	25.19 (3.53)	25.02 (3.43)
Anion Gap, mean (SD)	12.35 (4.12)	10.74 (3.38)	10.89 (3.74)
Creatinine (µmol/L), median [IQR]	72.00 [55.00, 100.50]	86.00 [70.00, 116.00]	71.00 [59.00, 89.00]
Vital Signs			
FiO_2_ (%), median [IQR]	28.00 [21.00, 82.50]	21.00 [21.00, 28.00]	21.00 [21.00, 21.00]
SpO_2_ (%), median [IQR]	94.00 [91.00, 96.00]	95.00 [93.00, 97.00]	96.00 [94.00, 98.00]
SpO_2_/FiO_2_,* median [IQR]	305.71 [109.85, 402.38]	447.62 [335.71, 457.14]	457.14 [442.86, 466.67]
Temperature (°C), mean (SD)	36.92 (0.31)	36.94 (0.54)	37.02 (0.53)
Systolic Blood Pressure (mm Hg), median [IQR]	127.00 [116.00, 143.50]	133.00 [118.50, 149.50]	123.00 [113.00, 137.00]
Diastolic Blood Pressure (mm Hg), median [IQR]	78.00 [70.50, 84.00]	74.00 [67.00, 80.00]	76.00 [68.00, 82.00]
Heart Rate (bpm), mean (SD)	99.53 (16.49)	87.10 (18.52)	95.89 (21.55)
Shock Index* median [IQR]	0.79 [0.64, 0.92]	0.66 [0.55, 0.75]	0.74 [0.63, 0.89]
Respiratory Rate (bpm), median [IQR]	26.00 [20.00, 30.00]	20.00 [20.00, 24.00]	20.00 [18.00, 20.00]
Medication			
Anticholesterolemic agents, *n* (%)	16 (20.3)	153 (55.6)	21 (9.9)
Antihypertensive agents, *n* (%)	19 (24.1)	181 (65.8)	46 (21.6)
Bronchodilator agents, *n* (%)	28 (35.4)	92 (33.5)	62 (29.1)
Diuretics, *n* (%)	17 (21.5)	105 (38.2)	15 (7.0)
Factor Xa Inhibitors, *n* (%)	1 (1.3)	45 (16.4)	4 (1.9)
Hypoglycemic agents, *n* (%)	33 (41.8)	145 (52.7)	39 (18.3)
Platelet aggregation inhibitors, *n* (%)	13 (16.5)	124 (45.1)	17 (8.0)
Imaging Data			
Opacities Numbers, *n* (%)			
0	0 (0.0)	33 (12.0)	117 (54.9)
1	0 (0.0)	59 (21.5)	33 (15.5)
2	76 (96.2)	155 (56.4)	55 (25.8)
3	3 (3.8)	28 (10.2)	7 (3.3)
4	0 (0.0)	0 (0.0)	1 (0.5)
Opacities Size (surface area %), mean (SD)	17 (8)	10 (8)	4 (6)
Clinical outcomes			
Length of stay (days), median [IQR]	14.99 [6.00, 34.42]	9.95 [4.61, 20.04]	5.04 [1.32, 11.51]
Mechanical ventilation, *n* (%)	19 (24.1)	27 (9.8)	2 (0.9)
ICU admission, *n* (%)	41 (51.9)	68 (24.7)	23 (10.8)
Death, *n* (%)	14 (17.7)	77 (28.0)	23 (10.8)
Other			
Wave (1st), *n* (%)	49 (62.0)	154 (56.0)	108 (50.7)

^†^ Those variables were excluded from the clustering algorithm effort because of the presence of other highly correlated variables.

^*^ Those variables were excluded from the clustering algorithm effort and computed after phenotypic classification for interpretability purposes.

Cluster 1 (*n* = 79, 14%) represented the group of patients with the most severe presentation having the highest NLR [median 9.9; interquartile range (IQR), 5.64 to 19.55], the highest rate of hypoxemia (median SpO_2_/FiO2 306; IQR, 109 to 402), and the highest radiographic burden, with 100% of patients with at least two pulmonary opacities and a mean total opacities size of 17% [standard deviation (SD), 8].

**Figure 3:**
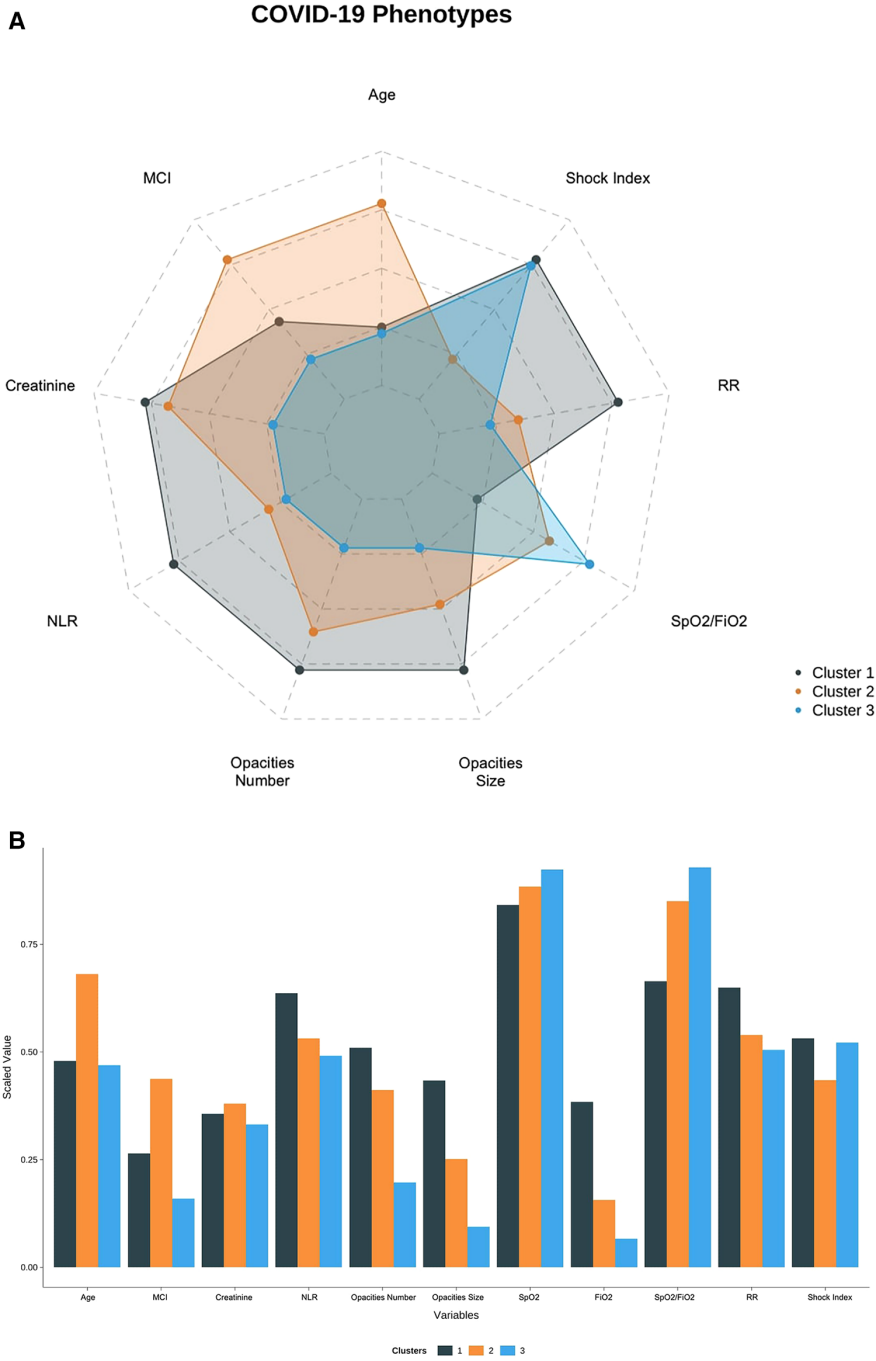
(**A**) Radar plot showing the distribution of clinical variables across clusters. (**B**) Bar Plot showing the distribution of clinical variables across clusters.

Cluster 2 (*n* = 275, 50%) and Cluster 3 (*n* = 203, 37%) were similar regarding their relatively non-severe clinical presentation but differed in terms of demographics. For Cluster 2, the median SpO2/FiO2 ratio was 447 (interquartile range, 335–457), and the proportion of patients with at least two pulmonary opacities was 66%. Cluster 2 also represented the oldest group (mean 75.4 years; SD 13) and the group with the highest proportion of comorbidities (mean MCI of 3.93; SD 1.72). Accordingly, Cluster 2 included patients with a high proportion of concurrent medication on admission:66% took antihypertensive agents, 57% hypolipemiant agents, 53% hypoglycemic agents, 45% antiplatelet agents, and 34% bronchodilator agents. For Cluster 3, the median SpO2/FiO2 was 457 (IQR, 443–467), and the proportion of patients with at least two pulmonary opacities was 30%. Cluster 3 represented the youngest cohort (mean 58 years; SD 19) and the group with the lowest comorbidities (mean MCI of 1.43; SD 1.50) (see Figures 3A,B).

### Phenotypes and clinical outcomes

Among the 547 patients in our study cohort, 436 were eligible for ICU admission and MV, as deemed by their POLST form, and were thus analyzed for clinical outcomes (see Figure 2).

**Figure 4:**
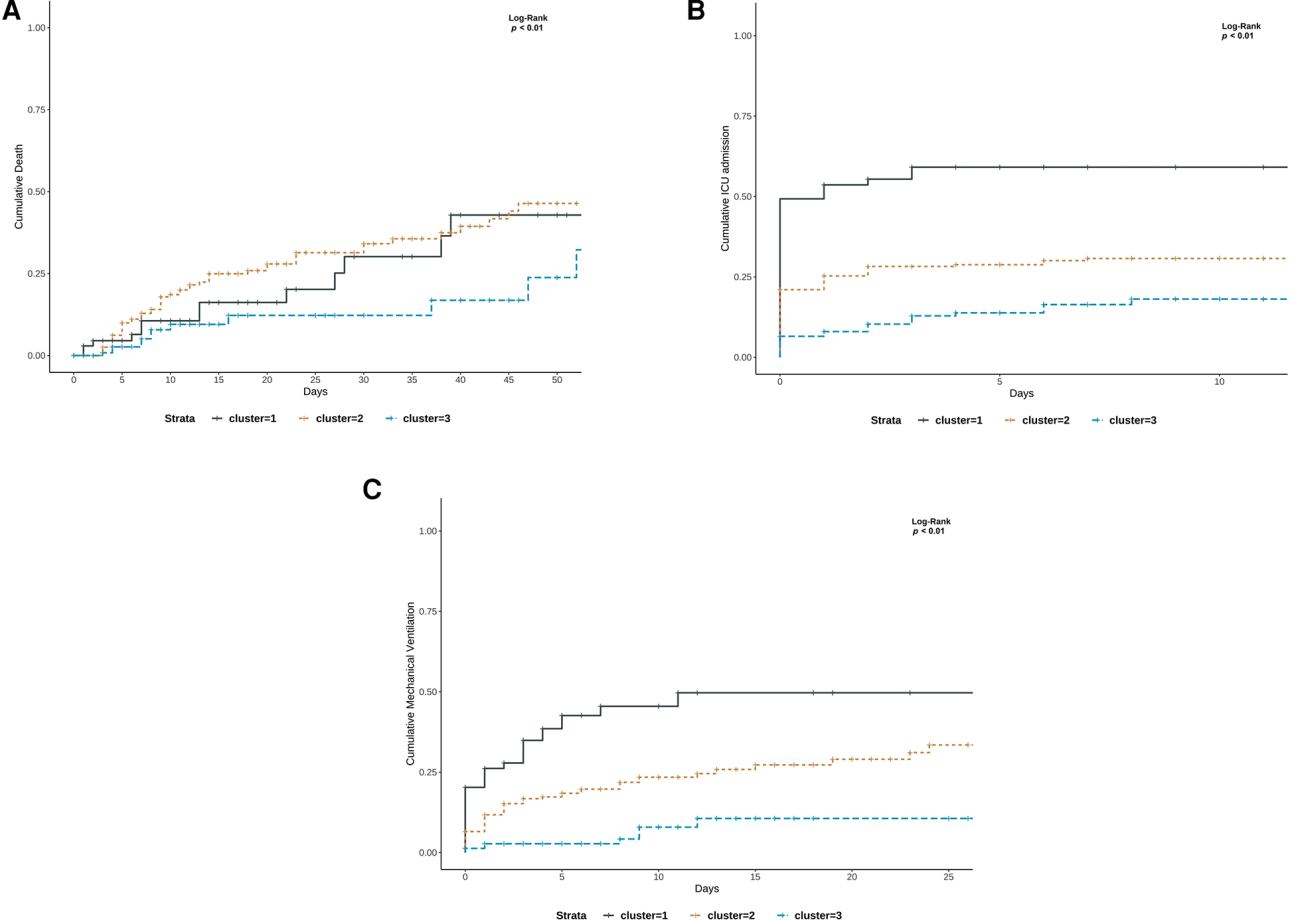
(**A–C**) Kaplan–Meier curves for clinical outcomes stratified by phenotypes: (**A**) death (**B**) ICU admission and (**C**) mechanical ventilation risk.

The cumulative mortality risk was significantly different across clusters (log-rank test, *p *= 0.01). The 30-day mortality risks were 30% (10%–45%, 95 CI), 34% (25%–42%, 95 CI), and 12% (3%–20%, 95 CI) for Clusters 1, 2, and 3, respectively (log-rank, *p* = 0.01). The cumulative ICU admission and mechanical ventilation risks were also statistically significant across all clusters (log-rank test, *p* ≤ 0.01 and *p* ≤ 0.01, respectively). More precisely, the 7-day ICU admission risk was 59% (45%–70%, 95 CI) for Cluster 1, 30% for Cluster 2 (24%–36%, 95 CI), and 26% (9%–23%, 95 CI) for Cluster 3. The 7-day mechanical ventilation risk was 45% (30%–57%, 95 CI) for Cluster 1, 20% (14%–25%, 95 CI) for Cluster 2, and 3% (0.5%–5%, 95 CI) for Cluster 3 (see Figure 4).

### Phenotypes interpretability and assignment

**Figure 5:**
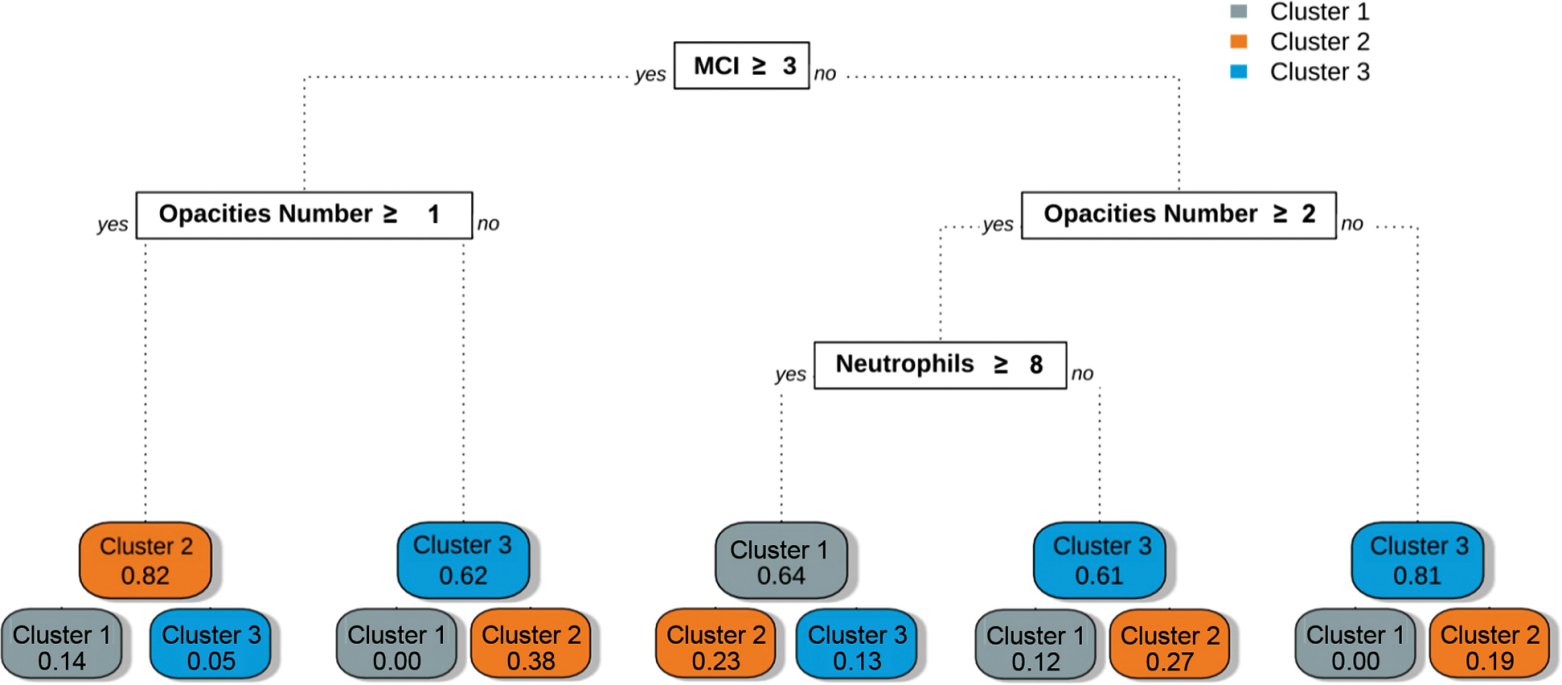
Phenotype assignment using decision tree-based rules. This graph represents the classification rules obtained after training the CART decision tree algorithm with the phenotypes as outcomes. The rules obtained could be used at the bedside to determine the phenotype of a given individual following simple steps. The rules are located in white square boxes, and each coloured node box displays the probability of the predicted class.

We developed a simple decision tree that allows patients to be assigned their respective clinical phenotypes using the rules shown in Figure 5. Using only three variables (MCI, opacities number, ANC) and following between two and three steps, one can assign a patient to one of the three phenotypes with a macro-averaged AUC of 0.84 (0.81–0.87, 95% CI) on our validation cohort. The other metrics are presented in Table 4. Subgroup analyses showed a consistent performance of the model across the first two waves of the pandemic. Detailed characteristics of our validation cohort are provided in Supplementary Table S4.

**Table 4:** Phenotype assignment model validation results.

Metrics	Result (Mean ± SD)*		
	Entire validation set (*n *= 135)	First wave (*n *= 68)	Second wave (*n *= 67)
AUC	0.84 ± 0.025	0.83 ± 0.058	0.85 ± 0.037
Accuracy	0.75 ± 0.037	0.75 ± 0.052	0.75 ± 0.053
Balanced accuracy	0.77 ± 0.030	0.77 ± 0.043	0.76 ± 0.042
Precision	0.69 ± 0.055	0.71 ± 0.077	0.69 ± 0.081
Recall	0.67 ± 0.043	0.68 ± 0.062	0.67 ± 0.060
F-1 score	0.66 ± 0.048	0.67 ± 0.063	0.66 ± 0.064

*The results presented were macro-averaged and validated on 1,000 bootstrap samples of the validation set.

### Phenotypes robustness: sensitivity analyses

When comparing the clustering results with and without the inclusion of imaging data, the adjusted Rand index was 0.18, indicating that the dissimilarity between the two clusterings was high. The average silhouette width also decreased after the removal of the imaging data (0.34 with imaging; 0.29 without imaging), indicating that clustering with the inclusion of imaging data yielded more homogeneous clusters.

**Figure 6:**
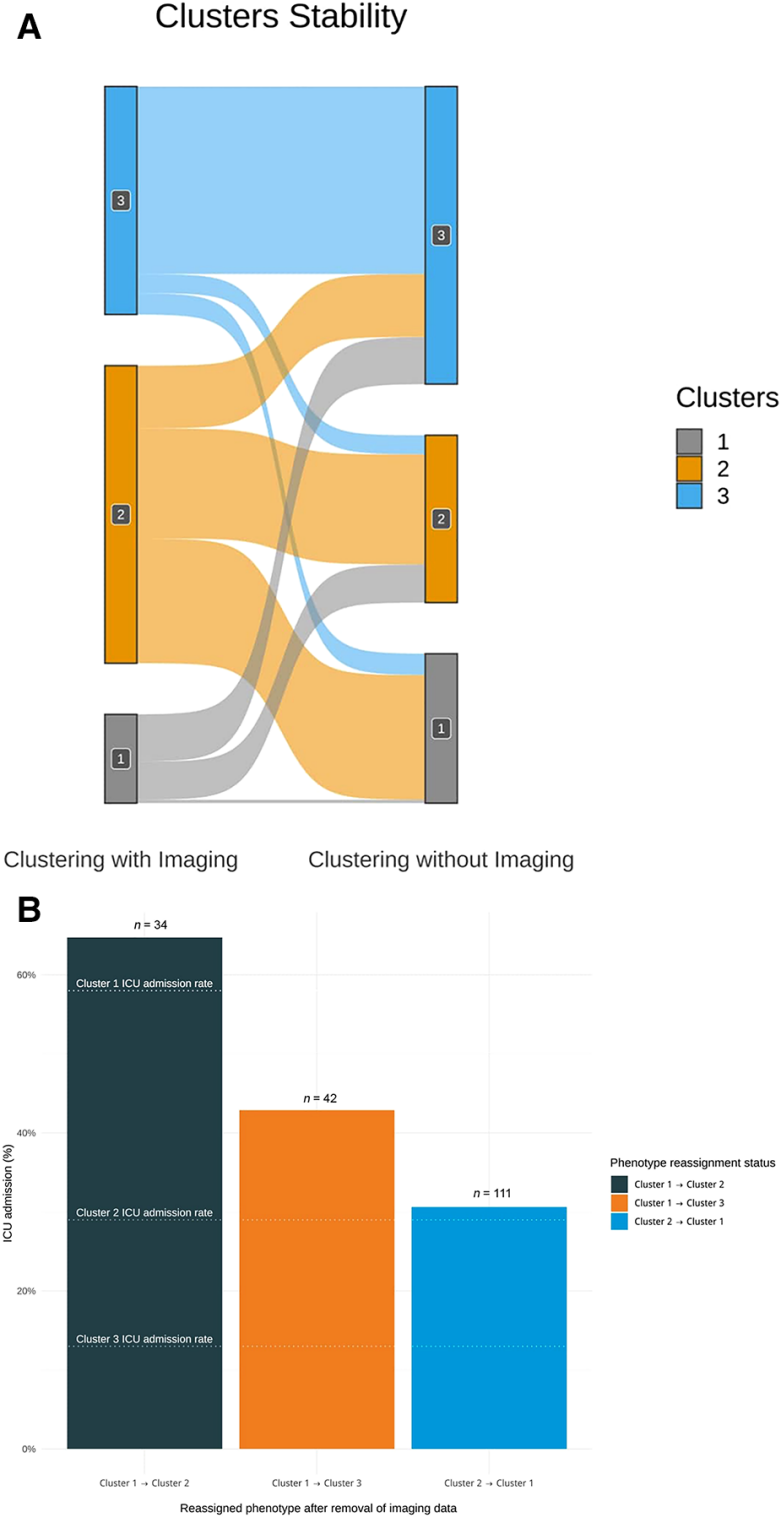
(**A**) Sankey diagram assessing clustering stability with and without imaging data. This plot shows the distribution of patients who underwent reclassification after the removal of imaging data in the clustering algorithm. A total of 279 observations were reclassified when imaging data were removed. A disproportionate number of reclassified observations originate from Cluster 1. 96% percent of the observations initially assigned to Cluster 1 (*n* = 76) were reclassified after the removal of imaging. As Cluster 1 is the most severe phenotype, the potential impact of this reclassification is not without consequences. (**B**) Bar plot showing the ICU admission rate of patients either unassigned from Cluster 1 or reassigned to Cluster 1 after removing the imaging data from the clustering effort. The graph qualitatively highlights that the phenotypic assignment is more clinically accurate with the inclusion of imaging data. After removing the imaging data, 34 patients were reassigned from Cluster 1 to Cluster 2 and 42 patients were reassigned to Cluster 3. The ICU admission rates of these two groups of patients were closer to that of their initial assigned phenotype. Likewise, the ICU admission rate of the 111 patients reassigned from Cluster 2 to Cluster 1 was closer to their initial phenotype. Knowing that Cluster 1 is the most severe phenotype, this suggests that the initial phenotypic assignment using imaging data was clinically appropriate.

We further characterized individuals who underwent phenotypic reclassification. 51 percent of the patients (*n *= 279) underwent reclassification (see Figure 6A), indicating that their phenotype changed after the removal of imaging data. The highest proportion of reclassified observations came from Cluster 1, as 96 percent of observations (*n *= 76) were reclassified to Clusters 2 or 3 after the removal of imaging data. The ICU admission rate of the 34 patients reassigned from Clusters 1 to 2 was 65%. The ICU admission rate of the 42 patients reassigned from Clusters 1 to 3 was 43%. Conversely, the 111 patients initially assigned to Cluster 2 and reassigned to Cluster 1 after removing the imaging data had an ICU admission rate of 30% (see Figure 6B).

## Discussion

We identified three clinical phenotypes with distinct clinical characteristics and outcomes using multimodal clinical data in patients admitted with COVID-19. The three phenotypes can be summarized as follows: severely hypoxemic with high radiological burden irrespective of age (Cluster 1), mildly hypoxemic with either a high comorbidity index or old age (Cluster 2), and mildly hypoxemic with a low comorbidity index (Cluster 3).

Although not primarily intended for use as a clinical prediction tool, the identified phenotypes had distinct clinical outcomes. Cluster 1 included patients with the most severe presentation and was thus, unsurprisingly, the phenotype with the highest ICU admission and mechanical ventilation risk.

Cluster 2 and Cluster 3 represented patients with similar milder clinical presentations but with distinct comorbidity profiles. Patients in Cluster 2 had a higher comorbidity index (3.93 vs. 1.43), and the 30-day mortality was higher than that in Cluster 3 (34% vs. 12%). Being able to distinguish phenotypes with apparent similar features but different outcomes is essential clinically. These represent patients currently treated identically, but who might benefit from a different and more targeted treatment approach.

When comparing our results with previous work (13–20), the number of clusters obtained was consistent, as all have identified three phenotypes. However, all cited studies have reported phenotypes suggesting a linear relationship between age and disease severity. This drastically differs from our findings, in which the most severe phenotype (Cluster 1) did not represent the oldest group. Our phenotypes reflect the complexity of the distribution of COVID-19 patients, in which age is not the sole determinant of severity. Further research is needed to understand the virological and immunological factors causing severe infection in this phenotype.

Despite having considered more than 30 variables in our clustering algorithm, only four subdomains were central to establishing the phenotypes: demographics, hematologic features, respiratory features, and imaging data. The impact of socio-demographics, comorbidities (39), and hypoxemia (40) on the clinical course of patients with COVID-19 has been well documented, and their relative importance in our clustering effort was thus expected. Furthermore, the neutrophil-to-lymphocyte ratio (NLR), previously identified as an independent risk predictor of disease severity in COVID-19 (41), was accordingly higher in Cluster 1. In contrast, mean platelet volume (MPV) did not significantly impact clustering results despite being associated with severe forms of the disease (42).

Our study emphasizes the importance of imaging data in COVID-19-related clustering. Through our sensitivity analysis, we showed that incorporating CXR enhanced the clinical value of the phenotypes. Even in the absence of ground truth in unsupervised machine learning, we showed that dismissing imaging data reduced the clinical accuracy of our clustering algorithm. A total of 111 patients initially assigned to Cluster 2 were reclassified into the more severe Cluster 1 after removing imaging data from the algorithm. We deemed the initial assignment to the less severe Cluster 2 accurately, given that the 30% ICU admission rate of these patients was lower than that of Cluster 1 (52%) and closer to that of Cluster 2 (25%). Similarly, 76 patients initially assigned to Cluster 1 were reclassified into Clusters 2 or 3 after the removal of imaging data. We deemed the initial assignment to severe Cluster 1 appropriate because the ICU admission rate of those patients (53%) exceeded those of Clusters 2 and 3 (25% and 11%, respectively) (see Figure 6B). The use of imaging helped correctly classify outlier patients as those with a lower or higher radiological burden than the majority of the patients in their respective phenotypic groups (see Supplementary Figures S4, S5).

Opacity size has not been previously used in other COVID-19 phenotyping studies. Instead, the number of opacities has been used as a proxy variable in only one other paper (13). The number of opacities is generally more accessible, as it can be directly extracted from CXR reports and does not require manual annotation of medical images. However, studies have shown that, although these two variables provide overlapping information, they are not interchangeable. Their respective values differ when predicting survival and the need for respiratory support in patients with COVID-19 (22).

Furthermore, automation of chest x-ray opacity annotation is increasingly feasible with publicly available deep-learning models that harmonize the process (25). We opted for manual annotation in this study, awaiting further validation of these tools for the COVID-19 population. However, chest x-ray annotation should not be viewed as a rate-limiting process. On the contrary, it should be encouraged in healthcare machine learning, as it allows for uniform inputs during training and upon model deployment (43).

Our study highlights the feasibility and importance of agnostic approaches to disease phenotyping with no *a priori* information about patient outcomes.

At the bedside, clinical phenotypes help categorize patients in an unbiased manner. Previous studies have shown that individual risk factors alone are insufficient to adequately stratify patients with COVID-19 (22). Thus, phenotypes offer a simple yet holistic means of describing patients with COVID-19 while incorporating clinical presentation and morbidity risk.

In clinical trials, phenotypes can help harmonize enrolled participants and facilitate the identification of patient subgroups benefiting from a given therapy. Recent clinical trials have revealed that distinct clinical presentations mandate distinct treatments, with some therapies benefiting only patients with severe diseases (44, 45). The inclusion criteria for these trials have made it difficult for clinicians to accurately identify patients who would benefit the most from these novel interventions. The controversy regarding the benefits of anticoagulation in critically and non-critically ill patients is a testimony to this observation (46). Using standardized phenotypes could eliminate the ambiguous nature of patient subgrouping and facilitate the comparison of outcomes across trials.

In the optics of a potential clinical implementation, we applied three aspects deemed key for the acceptance of machine learning algorithms in the clinical setting: usability, interpretability, and trustworthiness (47).

Usability—the extent to which an ML algorithm can be integrated efficiently in a healthcare environment—was achieved through our phenotypic assignment model. The variables needed to assign patients to phenotypes are readily available at the point of care, and phenotypic assignment can be performed following two to four rules (see Figure 5). We internally validated our model using a 25% holdout set with bootstrapping for the error estimation. Our model must be externally validated before use in clinical settings. Even though our patient population was diverse and representative of North American patients hospitalized with COVID-19, we recognize that the unicentric nature of our cohort limits the generalization of the phenotypes obtained. Nevertheless, the CART model remains helpful for demonstrating the applicability of such phenotypes in clinical settings.

Interpretability—the ability to understand the internal mechanism of an algorithm—in the context of clustering refers to providing intuition regarding how phenotypes differ. This was achieved through data visualization, as shown in Figures 3A,B serve as visual aids, highlighting the significant differences between the phenotypes. The CART assignment model also serves interpretability, as it gives clinicians insight into the workings of clustering, highlighting which features are most important in assigning phenotypes while also showing how decisions are made based on those features.

Trustworthiness, the ability to assess the validity and reliability of a machine learning output, is also provided through CART. The probabilistic nature of the algorithm allows for direct quantification of model uncertainty, as shown in Figure 5 (48). By assessing the probability distributions of each phenotype, clinicians can better appreciate the degree of confidence in a given phenotypic assignment.

We considered using other supervised learning models, but ultimately elected to use CART, as it offers a unique balance of overarching these three goals.

Finally, we recognized the discrepancy between our model's reported AUC and the other evaluation metrics (see Table 4). We believe that AUC is the most appropriate evaluation metric for the use case of our assignment model. The AUC requires predicted probabilities, whereas the F1-score requires predicted outcomes. Because our model's output is the probability distribution of phenotypes and not the predicted phenotype alone, the importance of the F1-score is secondary to that of the AUC. We also reported non-weighted macro-averaged validation metrics, which negatively affected other metrics.

Our study has some limitations. Clinical phenotypes do not offer a comprehensive explanatory model of observed disease heterogeneity (49). However, these studies lay the groundwork for understanding COVID-19 pathobiology. Studies linking biobank data to clinical phenotypes allow us to capture the taxonomic complexity of the disease and describe how phenotypes differ in terms of their pathogenic mechanisms (50, 51).

Multiple variables could not be included because they were either not captured in our electronic health record (e.g., time from onset of symptoms, mechanical ventilation parameters, and in-hospital complications) or excluded from our study because of missingness. However, missing values are common in clinical practice, and investigating risk stratification while considering the inherent characteristics of real-world data is important at the bedside (52). In addition, this enhances the applicability of our phenotypes, as they are based only on the most common variables available for patients admitted with COVID-19 (53). This differs from studies that have included flux cytometry and CD4+/CD8+ counts in their algorithms (14). In addition, the *omitted* variables do not seem to have significantly impacted our results, as the three clusters obtained were consistent with previous studies (13–19).

We also acknowledge that the use of our model was limited to the first 24 h of admission. We restricted our clustering effort to the data available within the first 24 h of admission to ease generalizability and to emulate the timing of triage decisions that are often made early upon admission. Although this methodological design choice serves its purpose, we recognize that phenotypes evolve over time (54). To expand the use of our model beyond the first 24 h of admission, we would need to repeat the clustering algorithm at different time points or use proper time-series clustering techniques, such as dynamic time warping. Given the inconsistent availability of imaging data after the first few days of admission, we did not consider these two options for this project.

Additionally, our study included patients admitted between January 1, 2020, and January 31, 2021, before the approval of most targeted therapies against COVID-19 and the mass vaccination campaign. Therefore, we did not assess the effect of vaccination, treatment, and variant type on phenotypes (21). Accordingly, this puts our algorithm at risk for a temporal dataset shift (55), and calibrating our clustering algorithm or applying the net reclassification index (NRI) will be necessary before exploiting it in the clinical setting. However, our work demonstrated the feasibility and potential clinical applicability of such methods to help identify patients at risk of clinical deterioration. Finally, because race-based data are not recorded in the Quebec healthcare system (56), we could not proceed to a sensitivity analysis according to race. For the same reason, we acknowledge that our work could be subject to algorithmic bias, as evidence has shown racial disparities in the clinical outcomes of patients with COVID-19 (57).

## Conclusion

We developed a multidimensional phenotypic analysis of COVID-19 patients and identified three distinct phenotypes, one specifically associated with worse clinical outcomes. Our study supports the feasibility of using real-world clinical data to conduct unsupervised phenotypic clustering while highlighting the importance of including imaging data in such endeavors. External validation and further research are needed to determine how phenotypes could impact clinical trial design and phenotype-guided treatment in clinical practice.

## Data Availability

The entire code, excluding the dataset, is publicly available on GitHub (https://github.com/CODA-19/models/tree/master/phenotyper). The data supporting this study's findings can be audited upon request from the corresponding author, MC, after appropriate privacy assessments and legal agreements in accordance with the Province of Quebec Privacy laws.
