# Supplementary material for: Interpretable clinical phenotypes among patients hospitalized with COVID-19 using cluster analysis
Source: Front Digit Health. 2023 Apr 11;5:1142822. doi: 10.3389/fdgth.2023.1142822 (PMC10128042; doi:10.3389/fdgth.2023.1142822)
Supplement: Supplementary file 1 [file Datasheet1.docx]

#### Supplementary Methods

##### Tabular data processing

We mapped all drugs into 73 recognized drug classes from the raw EHR database using the RxClass API (58). Similarly, ICD10 codes were mapped into comorbidities corresponding with Charlson’s score comorbidity index(59,60). All the variables of interest were sampled from the period of interest: the first 24 hours following a COVID-19-related admission. Because of the high frequency of the available data, we used the first value of the vital signs and laboratory values over the aforementioned period of interest. A COVID-19-related admission was defined as any COVID-19 hospitalization within seven days of a positive SARS-CoV-2 PCR test.

##### Image data annotation validation

Imaging data were obtained in the DICOM format, and frontal chest radiographs (posteroanterior and anteroposterior) were processed, discarding lateral chest radiographs. Images were manually annotated by a board-certified radiologist, and the total number of opacities and the relative total size of opacities for each radiograph.

To validate the annotation method, 240 images (42% of the dataset) were annotated twice by the same radiologist in a 3-month interval.  We calculated the interobserver coefficient for those two respective metrics. The results were a weighted Kappa coefficient of 0.621 for the number of opacities and an intraclass correlation (ICC) of 0.607 (p=0.012) for opacities size, corresponding to substantial agreement.

##### Clustering

Because multiple clustering approaches exist and the lack of consensus regarding the specific case of clustering mixed data type (categorical and continuous variable), we thoroughly compared numerous methods, including K-Means, PAM (61,62), Fuzzy and Agglomerative and Divisive Hierarchical clustering(63).

###### Data Transformation method

We compared two distinct approaches: the first, directly processing the variables to manually compute the Gower distance and generate our dissimilarity matrix (64). The second, using FAMD as a data reduction technique before computing the dissimilarity matrix.

This Gower distance metric was used as it is specifically suited for mixed data type datasets featuring both numeric and categorical variables. Specifically, non-normally distributed variables were appropriately addressed by log transformation. Continuous variables were range-normalized, and the distance was computed using the Manhattan distance. Ordinal variables were first ranked, and the Manhattan distance was subsequently used (the only ordinal variable in our dataset was the number of opacities, with 4 being the top-ranked variable). Nominal variables (binary variables) were converted into dummy variables, and the Dice coefficient was used as the distance metric (65). We did not assign different weights to the variables to minimize the amount of clinical input into the clustering effort.

The FAMD approach generates principal components and uses the projected coordinates as continuous variables that can subsequently be used in clustering algorithms. Similarly, before FAMD,  skewed continuous variables were log-transformed (skewness > 0.5), and highly correlated variables (correlation > 0.8) were excluded from the analysis. The only exclude variable based on high correlation was the total white blood cell count (WBC). This variable highly correlated with neutrophils (0.96).

Continuous variables were then scaled to unit variance. The categorical variables were transformed into a disjunctive data table (crisp coding) and scaled using the specific scaling of MCA (Multiple Correspondence Analysis). This approach balanced the influence of both continuous and categorical variables in the analysis. Subsequently, we generated a dissimilarity matrix using the Euclidean distance before evaluating multiple clustering algorithms.

The second approach was ultimately considered superior as deemed per various internal validation clustering metrics.

###### Comparing clustering methods

Many clustering algorithms exist, and the optimal method depends on the underlying nature of the data to which the algorithm is applied (66). In clinical medicine, clustering has proven to be challenging as integrating multimodal data requires careful preprocessing (67) and the mathematical notion of similarity when using mixed data types is heavily debated (68).

Using the FAMD data reduction method, we kept the first 14 components yielding an eigenvalue > 1 and a cumulative percentage of variance > 1 (see **S3 Table**).  We compared various partitioning and hierarchical clustering methods to determine the optimal clustering algorithm for our dataset. We used the optCluster package (31), an R package that allows the fast comparison of clustering algorithms for various numbers of k clusters. The four clustering algorithms compared were: K-means, PAM (partition around medoids), divisive, and agglomerative hierarchical clustering. We used three internal validation metrics and four cluster stability metrics to compare the performance of the aforementioned clustering algorithms.

As computed in the optCluster package, the internal validation metrics were connectivity, the Dunn index, and the average silhouette width. The stability measures used were the average proportion of non-overlap (APN), the average distance (AD), the average distance between means (ADM), and the figure of merit (FOM). The stability measures are a particular version of internal measures which evaluate the stability of a clustering result by comparing it with the clusters obtained by removing one column at a time. The optimal algorithm and the optimal number of clusters were then determined by the rank aggregation of the ranked lists of each validation metric using the weighted Spearman’s footrule distance and the cross-entropy Monte Carlo algorithm (69) .

###### Variable importance analysis (VIA)

We trained a Random Forest classifier using clusters as outcomes with all the candidate variables as predictors. We conducted a variable importance analysis to assess the relative importance of the variables in our clustering effort (33). We used the mean decrease in the Gini coefficient as the variable importance measure. The Gini coefficient is a measure of purity in tree-based classifiers that is low when the classification error rate at each split is low. The mean decrease in the Gini coefficient can thus be computed by assessing the overall performance of a random forest classifier after removing that variable. The higher the value of the mean decrease in the Gini coefficient, the higher the importance of the variable in the model. The variable importance plot shows the most discriminating features in descending order (**S2 Figure**).
