# Supplementary material for: Interpretable clinical phenotypes among patients hospitalized with COVID-19 using cluster analysis
Source: Front Digit Health. 2023 Apr 11;5:1142822. doi: 10.3389/fdgth.2023.1142822 (PMC10128042; doi:10.3389/fdgth.2023.1142822)
Supplement: Supplementary file 2 [file Datasheet2.docx]

**Supplementary Tables**

#### S1 Table. Complete baseline characteristics before data processing

#### S2 Table. Summary of clustering algorithms validation metrics results

#### S3 Table. Complete information regarding component analysis

#### S4 Table. Baseline characteristics in training and validation cohort

#### S5 Table. Phenotype assignment model training results

**Supplementary Figures**

##### S1 Figure. Clusters visualization on factor analysis of mixed data principal components plot

##### S2 Figure. Rank aggregation plot of the various clustering algorithms

##### S3 Figure. Variable Importance Plot (Mean decrease Gini)

##### S4 Figure. Factor analysis of mixed data individual plots highlighting misclassified observations when clustered without imaging data

##### S5 Figure. Opacity size density plots across the three clusters (obtained without initially using imaging data).

#### S1 Table. Complete baseline characteristics before data processing

| **Candidate variables** | **Missing (%)** | **Candidate variables** | **Missing (%)** | **Candidate variables** | **Missing (%)** |  |
| --- | --- | --- | --- | --- | --- | --- |
| **Demographics** | | **Laboratory Results** | | **Vital Signs** | |  |
| sex | 0 | amylase | 99.8 | fio2 | 24.1 |  |
| age | 0 | globulin | 99.5 | rr | 23.1 |  |
| weight | 76.1 | vitd | 99.3 | 97.9 | 24.2 |  |
| **Comorbidities (ICD code)** | | stab | 97.9 | dbp | 24.2 |  |
| ami | 73.3 | bili_direct | 95.4 | temp | 24 |  |
| chf | 73.3 | bili_indirect | 95.4 | so2 | 17.8 |  |
| pvd | 73.3 | protein | 92.2 | **Clinical Outcomes** | |  |
| cevd | 73.3 | ferritin | 90.5 | mechanical ventilation | 0 |  |
| dementia | 73.3 | ggt | 88.7 | icu admission | 0 |  |
| copd | 73.3 | pao2 | 88.5 | death | 0 |  |
| rheumd | 73.3 | ckmb | 88 | **Home Medication Data** | |  |
| pud | 73.3 | pt | 87.3 |  | 4.4 |  |
| mld | 73.3 | tsh | 83.9 |  |  |  |
| diab | 73.3 | fibrinogen | 80 |  |  |  |
| diabwc | 73.3 | bnp | 79.9 |  |  |  |
| hp | 73.3 | d_dimer | 78.8 |  |  |  |
| rend | 73.3 | ldh | 76.9 |  |  |  |
| canc | 73.3 | ck | 74.7 |  |  |  |
| msld | 73.3 | ast | 70.7 |  |  |  |
| metacanc | 73.3 | bun | 66.6 |  |  |  |
| aids | 73.3 | lactate | 64 |  |  |  |

**S2 Table.** Summary of clustering algorithms validation metrics results

|  | | **Number of clusters** | | | |
| --- | --- | --- | --- | --- | --- |
| **Clustering algorithm** | **Validity metric** | 3 | 4 | 5 | 6 |
| k-means | Connectivity | 233.6012 | 357.6246 | 507.9853 | 525.696 |
|  | Dunn | 0.1142 | 0.1237 | 0.1396 | 0.1108 |
|  | Silhouette | 0.1124 | 0.101 | 0.0831 | 0.0779 |
| pam | Connectivity | 5.8579 | 8.7869 | 11.7159 | 14.6448 |
|  | Dunn | 0.4616 | 0.48 | 0.4211 | 0.4115 |
|  | Silhouette | 0.3787 | 0.2906 | 0.2225 | 0.1913 |
| agnes | Connectivity | 477.244 | 599.6591 | 647.3206 | 640.6587 |
|  | Dunn | 0.0647 | 0.0647 | 0.0647 | 0.0871 |
|  | Silhouette | 0.0655 | 0.0528 | 0.0482 | 0.0506 |
| diana | Connectivity | 220.6798 | 246.9984 | 447.7028 | 466.6909 |
|  | Dunn | 0.1686 | 0.1746 | 0.1551 | 0.1671 |
|  | Silhouette | 0.1294 | 0.1151 | 0.0747 | 0.0754 |

Optimal scores

|  | Score | Method | Clusters |
| --- | --- | --- | --- |
| Connectivity | 5.8579 | agnes | 3 |
| Dunn | 0.4800 | agnes | 4 |
| Silhouette | 0.3787 | agnes | 3 |

#### S3 Table. Complete information regarding component analysis


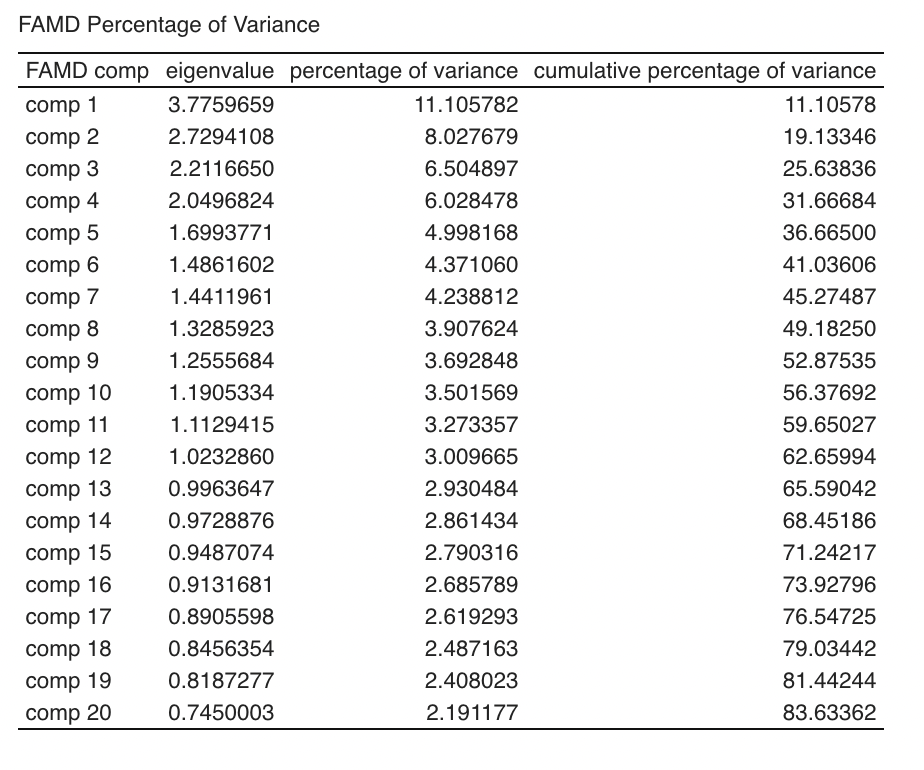


**S4 Table. Baseline characteristics in training and validation cohort**

|  | **Testing set *n=*135** | **Training test *n*=412** |
| --- | --- | --- |
| **Demographics** | | |
| **Age (years), mean (SD)** | 66.83 (17.12) | 66.48 (18.22) |
| **Sex (male), *n* (%)** | 75 (55.6) | 238 (57.8) |
| **Medicines Comorbidity Index (MCI), mean (SD)** | 2.70 (2.11) | 2.80 (2.03) |
| **Laboratory results** | | |
| **Hemoglobin, mean (SD)** | 126.54 (22.78) | 127.32 (20.45) |
| **Platelet, mean (SD)** | 193.00 [147.00, 278.50] | 208.50 [159.75, 271.00] |
| **WBC^†^, median [IQR]** | 6.70 [5.15, 9.75] | 6.80 [5.30, 10.03] |
| **Neutrophil count, median [IQR]** | 4.87 [3.60, 7.40] | 5.06 [3.61, 7.68] |
| **Lymphocyte count, median [IQR]** | 0.87 [0.58, 1.29] | 1.00 [0.66, 1.37] |
| **MCV, median [IQR]** | 90.00 [86.75, 94.40] | 89.80 [85.57, 93.70] |
| **MPV, mean (SD)** | 9.94 (1.28) | 9.87 (1.35) |
| **NLR****^*^, median [IQR]** | 5.30 [3.16, 11.33] | 5.16 [3.25, 9.84] |
| **Sodium, mean (SD)** | 137.84 (4.82) | 137.73 (5.12) |
| **Potassium, mean (SD)** | 3.99 (0.51) | 4.02 (0.53) |
| **Bicarbonate, mean (SD)** | 25.24 (3.74) | 24.85 (3.60) |
| **Anion Gap, mean (SD)** | 10.90 (3.47) | 11.10 (3.76) |
| **Creatinine (µmol/L) , median [IQR]** | 78.00 [58.50, 104.00] | 78.00 [64.00, 105.25] |
| **Vital Signs** | | |
| **FiO_2_ (%), median [IQR]** | 21.00 [21.00, 28.00] | 21.00 [21.00, 28.00] |
| **SpO_2_ (%), median [IQR]** | 96.00 [94.00, 97.00] | 95.00 [93.00, 97.00] |
| **SpO_2_/FiO^*^_2_, median [IQR]** | 447.62 [326.79, 459.52] | 450.00 [342.86, 461.90] |
| **Temperature (°C), mean (SD)** | 37.04 (0.51) | 36.95 (0.52) |
| **Systolic Blood Pressure (mm Hg) , median [IQR]** | 132.00 [116.50, 149.00] | 129.00 [116.00, 143.00] |
| **Diastolic Blood Pressure (mm Hg) , median [IQR]** | 75.00 [68.50, 82.00] | 75.00 [67.00, 82.00] |
| **Heart Rate (bpm)****, mean (SD)** | 90.79 (20.08) | 92.70 (20.29) |
| **Shock Index^*^, median [IQR]** | 0.67 [0.57, 0.78] | 0.70 [0.58, 0.83] |
| **Respiratory Rate (bpm) , median [IQR]** | 20.00 [18.00, 24.00] | 20.00 [20.00, 24.00] |
| **Medication** | | |
| **Anticholesterolemic agents, *n* (%)** | 39 (28.9) | 143 (34.7) |
| **Antihypertensive agents, *n* (%)** | 58 (43.0) | 178 (43.2) |
| **Bronchodilator agents, *n* (%)** | 47 (34.8) | 126 (30.6) |
| **Diuretics, *n* (%)** | 32 (23.7) | 103 (25.0) |
| **Factor Xa Inhibitors, *n* (%)** | 17 (12.6) | 31 ( 7.5) |
| **Hypoglycemic agents, *n* (%)** | 45 (33.3) | 165 (40.0) |
| **Platelet aggregation inhibitors, *n* (%)** | 35 (25.9) | 115 (27.9) |
| **Imaging Data** | | |
| **Opacities Numbers, *n* (%)** | | |
| **0** | 37 (27.4) | 104 (25.2) |
| **1** | 18 (13.3) | 71 (17.2) |
| **2** | 73 (54.1) | 206 (50.0) |
| **3** | 7 ( 5.2) | 30 ( 7.3) |
| **4** | 0 ( 0.0) | 1 ( 0.2) |
| **Opacities Size (surface area %), mean (SD)** | 0.09 (0.09) | 0.09 (0.08) |
| **Clinical outcomes** | | |
| **Mechanical ventilation, *n* (%)** | 15 (11.1) | 33 ( 8.0) |
| **ICU admission, *n* (%)** | 34 (25.2) | 98 (23.8) |
| **Death, *n* (%)** | 33 (24.4) | 80 (19.4) |
| **Phenotypes** | | |
| Clusters, *n* (%) | | |
| Cluster 1 | 19 (14.1) | 60 (14.6) |
| Cluster 2 | 66 (48.9) | 199 (48.3) |
| Cluster 3 | 50 (37.0) | 153 (37.1) |
| **Other** | | |
| **Wave (1st) , *n* (%)** | 77 (57.0) | 218 (52.9) |

**S5 Table. Phenotype assignment model training results (***n* = 438)

|  | **Metric (%)** | | | |
| --- | --- | --- | --- | --- |
| **Hyperparameter (Cp)** | Accuracy | Balanced accuracy | F-1 score | AUC |
| 0.023 | 72 | 74 | 66 | 76 |
| 0.028* | 69 | 73 | 64 | 77 |
| 0.042 | 67 | 72 | 63 | 73 |
| 0.056 | 64 | 65 | - | 65 |
| 0.357 | 37 | 43 | - | 27 |

The table shows the performance of the CART model on the training set for different values of the Complexity parameter.

* The final model used a value of Cp = 0.024

##### S1 Figure. Clusters visualization on factor analysis of mixed data principal components plot


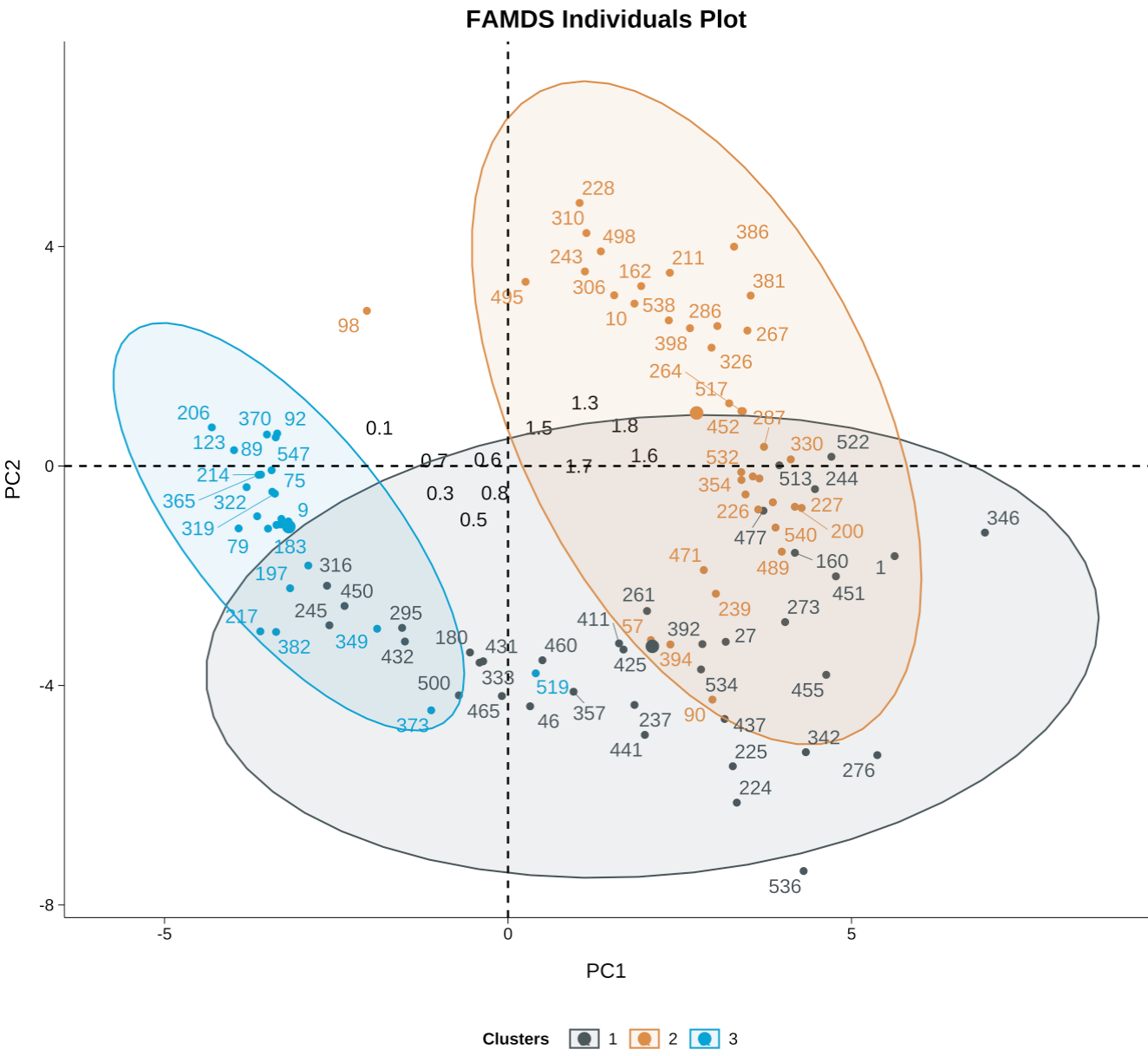


##### S2 Figure. Rank aggregation plot of the various clustering algorithms

#####
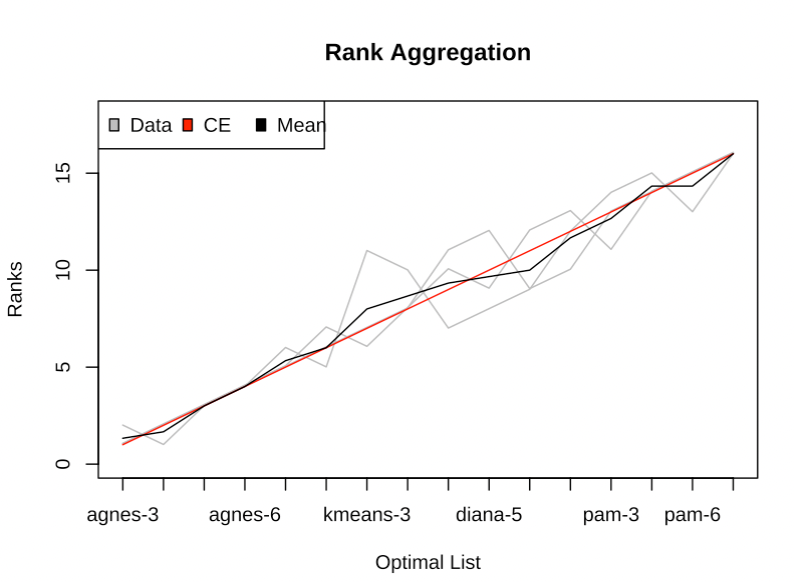


##### The bottom plot shows the individual ranks of the clustering algorithms from the data (in grey), the final "optimal" rankings (in red), and the average ranking of each clustering algorithm (in black).

##### S3 Figure. Variable Importance Plot of the variables used in the clustering algorithm


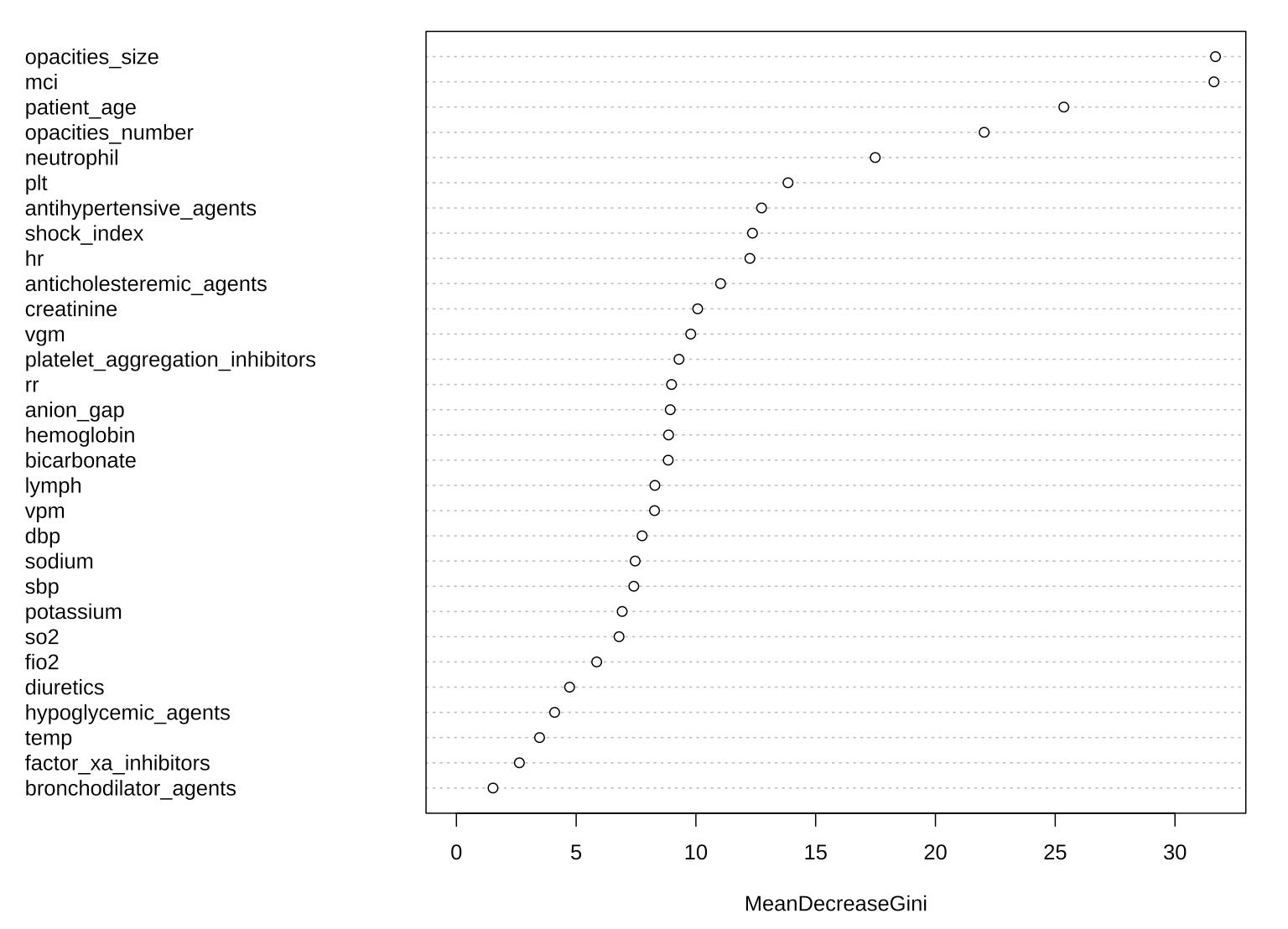


The higher the value of mean decrease accuracy or mean decrease Gini score, the higher the importance of the variable in the model.

##### S4 Figure. Factor analysis of mixed data individual plots highlighting misclassified observations when clustered without imaging data


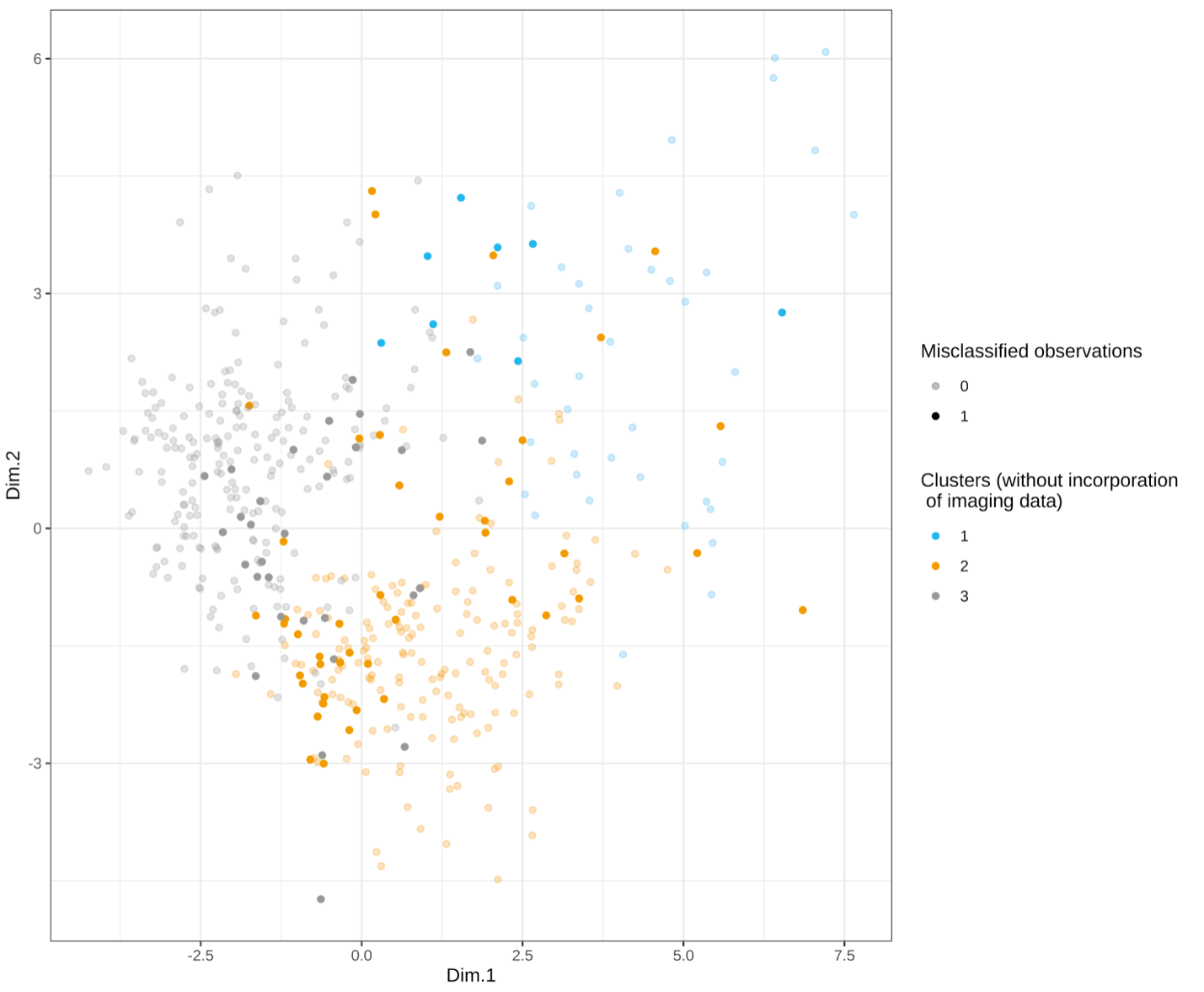


This FAMD plot highlights patients who underwent reclassification when removing imaging data in the clustering effort. Visually, those patients represent a group of observations at the frontier of different clusters.  These observations thus particularly benefit from additional imaging data to increase the reliability of their respective cluster assignments.

##### S5 Figure. Opacity size density plots across the three clusters after removing imaging data


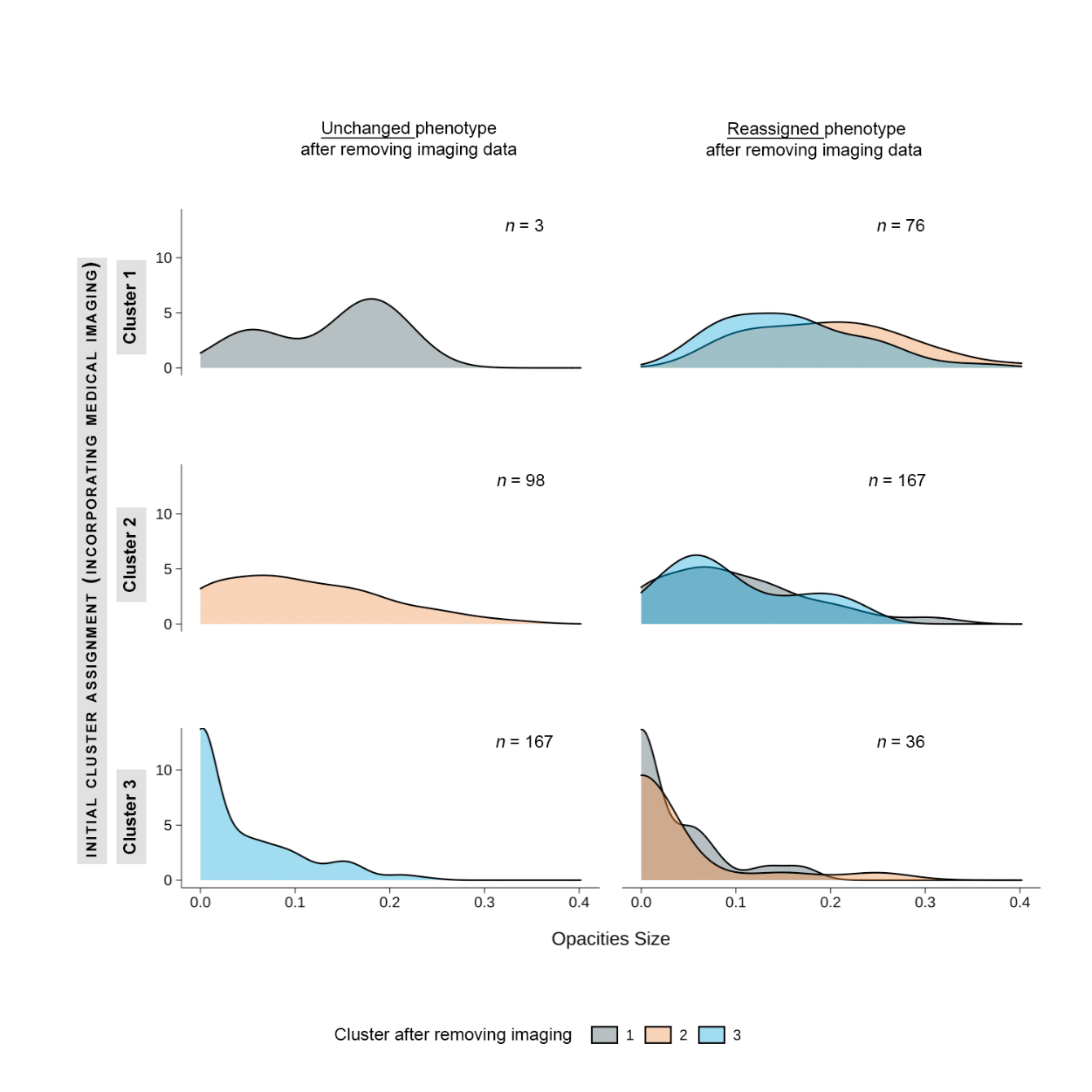


This plot highlights the opacity size of the reclassified observations after removing imaging data. For each cluster, the first column shows the distribution of opacities size of the observations for which the assigned cluster remained unchanged. In contrast, the second column shows the distribution of the observations that underwent phenotypic reclassification after removing imaging.

76 patients initially assigned to Cluster 1 were *misclassified* to Cluster 2 or 3. Since Cluster 1 represents the most severe phenotype, reclassifying those patients was deemed inappropriate. The distribution of the opacities size of the reclassified observations showed a relatively significant burden of pulmonary infiltrates that is incompatible with the less severe clusters Cluster 2 and 3.

Similar inferences can be made for the reclassified observations from Cluster 2 to Cluster 1 or 3 and reclassified observations from Cluster 3 to 1 or 2.

For a subgroup of patients, imaging is critical to discriminate between phenotypes appropriately.
